# Vitamin D Levels in Sows from Five Danish Outdoor Herds

**DOI:** 10.3390/ani12030299

**Published:** 2022-01-26

**Authors:** Sine Stricker Jakobsen, Jette Jakobsen, Jens Peter Nielsen

**Affiliations:** 1 Department of Veterinary and Animal Sciences, University of Copenhagen, 1870 Frederiksberg C, Denmark; sine.stricker.jakobsen@sund.ku.dk (S.S.J.); jpni@sund.ku.dk (J.P.N.); 2 National Food Institute, Technical University of Denmark, 2800 Kgs. Lyngby, Denmark

**Keywords:** vitamin D_3_, 25-hydroxyvitamin D_3_, sows, vitamin D status

## Abstract

**Simple Summary:**

A cross-sectional study on vitamin D_3_ status was conducted in five Danish outdoor sow herds throughout August 2020. The aim was to determine the vitamin D status of outdoor sows during the peak sunshine season. The average 25-hydroxyvitamin D_3_ concentration in serum was 67 ± 16 ng 25(OH)D_3_/mL in outdoor sows, which is considerably higher than levels found in sows housed indoors and fed a standard diet supplemented with vitamin D.

**Abstract:**

Vitamin D is essential for sow health and productivity. Standard sow feed is therefore supplemented with vitamin D_3_ or 25-hydroxyvitamin D_3_ (25(OH)D_3_). However, it is uncertain whether the levels achieved are adequate for optimal performance. Currently, information on serum levels of vitamin D in pigs reared under both indoor and outdoor conditions is lacking. In August 2020, we obtained blood samples from 97 organic newly weaned sows housed outdoors during pregnancy and farrowing and used these to test for vitamin D in serum. The average concentration was 67 ± 16 ng 25(OH)D_3_/mL with a range of 32 to 134 ng 25(OH)D_3_/mL. The vitamin D_3_ content was 21 ± 7 ng/mL, ranging from 9 to 48 ng/mL. The average number of hours of sun from June to August was 7.0 ± 0.5 h/day. Parity, farm and body condition score did not significantly affect serum levels of 25(OH)D_3_.

## 1. Introduction

Vitamin D is essential for a number of body functions including bone formation, immune system function, reproductive performance and growth [1,2,3,4]. Vitamin D is hydroxylated in the liver to form 25-hydroxyvitamin D (25(OH)D), which is the accepted biomarker for vitamin D status. In humans, reference values for 25(OH)D concentration in serum are still debated, but recommendations from both the Danish and the American health agencies agree that concentrations above 20 ng/mL are sufficient for humans in terms of bone health and general health in healthy individuals [5,6]. Likewise, humans with 25(OH)D concentrations below 20 ng/mL are considered to be vitamin D insufficient, while those with concentrations below 12 ng/mL are considered deficient. No such definitions of vitamin D insufficiency have been established for pigs. Pigs in indoor production systems do not produce vitamin D_3_ in their skin since they are not exposed to ultraviolet B (UVB, 280–315 nm) light from the sun or other sources. Therefore, they solely rely on vitamin D in feed to meet their nutritional requirements. It is not known whether a sufficient vitamin D serum concentration is obtained in indoor production and whether increased vitamin D levels would result in heavier piglets at birth, better reproduction and improved growth [3,7,8,9,10,11]. European legislation includes provisions on the maximum allowable amount of added vitamin D per kg of dry matter for pigs [12]. Vitamin D toxicity can lead to anorexia, vomiting, calcification of soft tissue, weight loss, lethargy and eventually death [13,14,15,16]. Vitamin D_3_ produced in the skin after sun exposure will, however, never reach toxic levels since excess vitamin D_3_ is degraded by sunlight [17]. This means that pigs exposed to natural sunlight for a prolonged period of time are expected to have high—though not toxic—levels of vitamin D. Since information about vitamin D serum levels in sows from outdoor herds is very sparse, it is also difficult to set target values for sows in indoor production. This inspired us to investigate the vitamin D levels in serum from Danish organic sows housed outdoors during the summer.

## 2. Materials and Methods

### 2.1. Experimental Design

The study was set up as a cross-sectional study to estimate the level of vitamin D in serum from newly weaned sows in organic Danish herds located at 55–57° N during the summer of 2020. We chose to sample in August because high vitamin D levels were observed in humans in Denmark during this month [18].

A sample size calculation to estimate the mean within a population was calculated using the formula$\;n=\frac{Z_{1-\alpha /2}^{2}\sigma^{2}}{L^{2}}$ where $Z_{1-\alpha /2}^{2}$ is the value of the standard normal distribution, σ is the standard deviation and L is the maximum allowable error [19]. Based on a standard deviation of 15 [20], an allowable error of 3 and a population size of 8500 (where population size was the estimated number of sows in Danish organic outdoor herds with 150 sows or more [21]). Sample size (*n*) was adjusted for population size creating the final sample size (*n*_a_) by using the equation *n*_a_ = $\frac{n}{1+\frac{n}{N}}$, this resulted in a sample size of 95.

### 2.2. Animals and Farms

Seven veterinary pig practices were contacted in order to identify farms willing to participate in the project. Veterinarians from three different veterinary practices were each able to find 1–2 farms willing to participate, of which five farms agreed to participate. Twenty sows were sampled from each farm except for farm D, which only had 14 newly weaned sows on the day of sampling, and farm E, from which three extra samples were obtained to compensate for the samples that were not obtained on farm D.

Sows were sampled within 2 days after weaning. Only one farm was sampled per day. All sows had spent at least 23 weeks in outdoor conditions prior to sampling. Sows were selected by convenience. If sows were housed in more than one pen, then samples were obtained from all pens. All sows were given a body condition score where 1 was thin, 2 was average and 3 was fat. Four of the farms were also able to provide information about the parity of the included animals. Sows were either Danish Landrace and Yorkshire cross from Danbred [22] or TN70 from Topigs Norsvin [23]. Breed information for each sow was not included as a factor in the statistical analysis as not all farms were able to provide this information.

**Figure 1:**
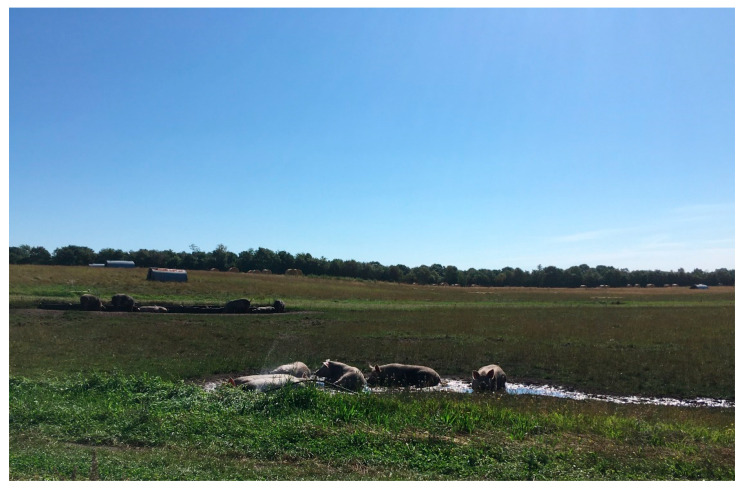
Organic outdoor gestating sows resting in their mud hole on a sunny day.

Organic pig producers of Denmark have to comply with a comprehensive set of rules in order to maintain their status as organic farmers [24]. Organic sows must be housed outdoors during all seasons. During gestation, sows are usually housed in small groups with a shared shed to provide them some protection from the weather, see Figure 1. They must also have access to a mud hole in order to perform their natural mud bath behavior in the hot season. During lactation, sows are most often housed individually with their litters in farrowing huts and with access to mud bathing.

Danish recommendations for vitamin D_3_ in pregnant and lactating sows is 800 i.u./Feed unit sow. For digestible phosphorus, the recommendations are 2.0 g/Feed unit sow during pregnancy and 3.0 g/Feed unit sow during lactation while for digestible calcium, these values are 7.0 g/Feed unit sow in herds not using phytase during pregnancy and 8.0 g/Feed unit sow during lactation [25].

### 2.3. Sun Exposure

Data on the hours of sunlight per month were obtained from the Danish Meteorological Institute (DMI) weather stations closest to each of the farms [26,27,28]. DMI has measuring stations throughout Denmark, and although Denmark only covers a small geographical area, data from the station closest in proximity to each farm were added to our data to provide information about sun exposure during the sampling period. Two of the farms were located in close proximity to each other and therefore share data from the same weather station. 

### 2.4. Sampling of Blood and Serum

Blood samples were obtained by restraining the sow using a snout snare and puncturing the jugular vein using a needle of 18 G × 1.5 BD ref 360,748 (Becton Dickinson, Franklin Lakes, NJ, USA), a 10 mL dry tube BD ref 367,896 and a vacutainer holder. Blood samples were stored at room temperature for 30 min and at 5 °C for 6–24 h, centrifuged for 15 min at 2500× *g* before the serum carefully transferred to a separate vial. The serum samples were stored at −80°C until analysis they were analyzed within 6 weeks.

### 2.5. Vitamin D Analysis

Serum samples were analyzed using a method described in detail elsewhere [29]. In short, 100 µL of serum was added internal standards (80 ng ^13^C-vitamin D_3_ and ^13^C-25(OH) D_3_) followed by precipitation of the protein by use of 300 µL acetonitrile. Then the solution was cleaned up by HybridSPE and acetonitrile as eluent. After evaporation, the extract was derivatized by 188 µg 4-phenyl-1,2,4-triazoline-3,5-dione for five minutes in the dark. Separation was performed on an Agilent 1200 Series HPLC mounted with a C18-column, combined with a gradient of methanol:water added ammonium formate as additive, and quantification of 25(OH)D_3_, 25(OH)D_2_, vitamin D_3_ and vitamin D_2_ were performed on an Agilent 6470 Triple Quadrupole MS equipped with a Jet Stream ion source (Agilent Technologies, Santa Clara, CA, USA). All samples were analyzed in duplicate, and a house reference sample was included in each series to ensure validity. The house reference sample was measured against a certified reference sample.

### 2.6. Statistical Analysis

The statistical software Rstudio (Rstudio, Inc., Boston, MA, USA) was used to analyze the data.

Analysis of variance (ANOVA) was used to check for differences in 25(OH)D_3_ and vitamin D_3_ levels across farms/sampling dates.

Data on 25(OH)D_3_ and vitamin D_3_ levels were checked for normal distribution and equal variances. Data were log-transformed in order to obtain normal distribution, and a linear mixed model was run for both 25(OH)D_3_ and vitamin D_3_, with farm as a random effect and body condition score and grouped parity (sows of parity 1–3 categorized as “young” and sows of parity 4–8 as “old”) as fixed effects.

Farm D was excluded from the model since they could not provide information on parity. 

## 3. Results

The mean 25(OH)D_3_ serum concentration for all samples was 67 ± 16 ng/mL, ranging from 32 ng/mL to 134 ng/mL. The mean level of vitamin D_3_ was 20 ± 7 ng/mL, ranging from 9 ng/mL to 48 ng/mL (Table 1).

There was no significant difference in average 25(OH)D_3_ serum concentrations among farms. The model showed no significant effect of parity or body condition on 25(OH)D_3_ serum levels. 

A significant difference (*p* < 0.05) was found in vitamin D_3_ serum levels among farms. The model showed no significant effect of parity or body condition on vitamin D3 levels.

The levels of 25(OH)D_2_ and vitamin D_2_ were insignificant, thus no statistical tests were performed for any differences between the farms.

## 4. Discussion

To our knowledge, this is the first study to investigate vitamin D status in Danish outdoor sows. The results illustrate a mean level of 25(OH)D_3_ of 67 ± 16 ng/mL, which is in agreement with the levels (57.2 ± 8.9 ng/mL) observed in outdoor sows in a survey conducted in the Upper Midwest of the United States of America in June 2011 [30].

Neither body condition score, parity, nor farm/sampling date significantly affected the levels of 25(OH)D_3_ in serum in this study. The sampling date could possibly affect the outcome since there is a decline in UVB exposure from the sun in August [31]. However, all samples were collected within a period of 18 days in August, and no effect of sampling date was observed. We report the hours of sun during the 2½–3 months before sampling, as any hours of sun prior to this would have no additional effect due to the half-life of 25(OH)D. In humans, the half-life is 14–21 days, but this remains unknown for pigs [32]. Since age is a factor that can affect vitamin D production in humans [33], the parity of the sows was registered and used in the model as a proxy for age. However, no effect of parity was observed, which may be due to the fact that the maximum parity was 8, corresponding to approximately 4 years of age. 

Serum levels for both 25(OH)D_3_ and vitamin D_3_ showed high within-herd variation, supporting previous findings in studies of indoor pigs exposed to UVB light, which also demonstrated high individual variability in serum levels [20,29].

Vitamin D_3_ levels were significantly different across farms and sampling dates. Vitamin D_3_ has a shorter half-life than 25(OH)D_3_ and therefore variations in sun exposure would be expected to affect the vitamin D_3_ levels more rapidly. The contribution of 25(OH)D_2_ and vitamin D_2_ to the total 25(OH)D and vitamin D levels was very low at <2%.

Levels of 25(OH)D_2_ were on average 1.0 ± 0.3 ng/mL, which contribute insignificantly (<1.5%) to the total vitamin D status. The content of 25(OH)D_2_ is expected to derive from metabolized vitamin D_2_ in the grass/straw eaten by the sows [34].

Sows in this study were housed outdoors prior to sampling and were thereby exposed to sunlight. However, we do not know exactly how many hours of UVB the sows would have had on a daily or monthly basis. Sows were free to immerse themselves in mud baths, seek shade, or stay in their huts, all of which would decrease sun exposure and thereby vitamin D_3_ production.

Danish sow diet recommendations include 800 IU Vitamin D_3_/kg feed [25]. Sows housed indoors receiving a diet containing 800 IU vitamin D_3_/kg were reported to have a serum 25(OH)D_3_ level of 30–35 ng/mL at the time of weaning [35,36]. Levels in this study were almost twice as high as the levels in indoor sows at the time of weaning.

Sufficient levels of 25(OH)D in serum were established within human medicine, although these are still debated from time to time. The Danish Health Authority considers 25(OH)D serum levels above 50 nmol/L to be sufficient [37]. However, there are no such established levels for pigs. It would be beneficial to ascertain whether serum levels of 25(OH)D could be useful in determining whether sows have a sufficient supply of vitamin D. Establishing vitamin D levels obtained from sows housed under outdoor conditions could be the first step to a better understanding of optimal vitamin D levels for pigs. Samples from this study could serve as a reference for newly weaned sows housed outdoors, with a reference interval of 35–99 ng/mL, which is the 95% interval for the vitamin D status in the 97 sows.

We investigated the vitamin D status in free-range sows at the time of expected highest level, i.e., July/August, but we have no data at the expected lowest level, i.e., February/March [18]. However, we do have information of such comparison for the content of 25(OH)D_3_ and vitamin D_3_ in shoulder meat from Danish, free-range pigs slaughtered in August (2018) and March (2019) [38]. The content of 25(OH)D_3_ and vitamin D_3_ in subcutaneous fat from shoulders from August and from March was significantly different (*p* < 0.001). In the shoulders from August compared to those from March, the content was five times higher for 25(OH)D_3_ and 11 times higher for vitamin D_3_. Based on those results, we presume that vitamin D status in free-range pigs in Denmark in March will be at least five times lower compared to our results reported for August. Further studies to characterize the serum status of outdoor sows during the winter season in order to understand seasonality and possibly the half-life of 25(OH)D_3_ in sows are needed.

## 5. Conclusions

We determined the vitamin D status of 97 organic newly weaned sows housed outdoors for more than 23 weeks in Denmark (55° N). The average serum concentration of Vitamin D3 was 67 ± 16 ng 25(OH)D_3_/_mL_ serum. Parity and body condition score did not significantly affect serum levels of 25(OH)D_3_. The sampling was performed in August, which was expected to generate the highest vitamin D status during summer. The average number of hours of sun over the 3 months prior to sampling was 7.0 ± 0.5 h/day. The results could serve as a reference for newly weaned sows housed outdoors with a reference interval of 35–99 ng/mL (95% range).

## Figures and Tables

**Table 1:** Number of sows from each farm (A–F), mean values and standard deviations for 25(OH)D_3_, vitamin D_3_, 25-hydroxyvitamin D_2_ (25(OH)D_2_), parity and body condition score. Sun exposure in June, July and August: daily (hours/day) and total hours.

	All	Farm A	Farm B	Farm C	Farm D	Farm E
Number of sows (n)	97	20	20	20	14	23
25(OH)D_3_ (ng/mL)	67 (16)	71 (15)	71 (19)	65 (14)	70 (20)	61 (14)
Vitamin D_3_ (ng/mL)	20 (7)	28 (7)	23 (5)	16 (4)	23 (7)	15 (3)
25(OH)D_2_ (ng/mL)	1.0 (0.3)	1.1 (0.3)	1.0 (0.3)	0.9 (0.2)	1.2 (0.6)	1.1 (0.3)
Vitamin D_2_ (ng/mL)	0.3 (0.1)	0.4 (0.1)	0.3 (0.1)	0.2 (0.0)	0.2 (0.1)	0.2 (0.0)
Parity	2.5 (1.6)	3.1 (1.8)	2.6 (1.8)	2.0 (0.9)	-	2.3 (1.6)
Body condition	1.7 (0.6)	1.4 (0.5)	1.8 (0.6)	2.0 (0.2)	2.1 (0.7)	1.7 (0.7)
Sun exposure June–August Hours/day *	7.0 (0.5)	7.2	6.7	7.0	8.2	7.0
Total hours	554 (65)	502	477	543	657	612

- no information available. * Total hours of sun was adjusted according to the date of sampling in August, so farms sampled early in August had fewer hours of sunshine in August than farms sampled later. Two farms (C and E) share data from the same weather measuring station.

## Data Availability

The data presented in this study are available on request from the corresponding author. The data are not publicly available due to privacy restrictions.
